# Supplementary material for: Moss enables high sensitivity single-nucleotide variant calling from multiple bulk DNA tumor samples
Source: Nat Commun. 2021 Apr 13;12:2204. doi: 10.1038/s41467-021-22466-9 (PMC8044184; doi:10.1038/s41467-021-22466-9)
Supplement: Supplementary file 1 — Supplementary Information [file 41467_2021_22466_MOESM1_ESM.pdf]

Supplementary Material for "Moss Enables High Sensitivity Single-Nucleotide Variant Calling from Multiple Bulk DNA Tumor Samples"

Chuanyi Zhang, Mohammed El-Kebir, Idoia Ochoa

Contents

A Additional results **2**

B Implementation details **13**

C Commands **15**

C.1 Strelka2 15
C.2 Mutect2 15
C.3 Moss 15
C.3.1 Installation 15
C.3.2 Install Moss via Conda 16
C.3.3 Build Moss from source 16
C.4 Usage 16
C.4.1 Docker usage 17

List of Figures

S1 Precision-recall curves on simulated data 4
S2 Comparison of run time on simulated dataset 5
S3 Results of Moss with Strelka2 on the HCC dataset 6
S4 Distribution of base qualities of the missed SNV in three samples 7
S5 Common SNVs called by Mutect2 and Moss per quadrants in the HCC dataset. 7
S6 Scatter plots of the number of supporting samples and the VAF in the normal sample 8
S7 Manual review annotations 8
S8 Results of Moss with Mutect2 on downsampled HCC data. 9
S9 Comparison of run time and memory usage on HCC dataset 10
S10 Distribution of base qualities of the missed SNV in AML dataset 10
S11 Common SNVs called by Mutect2 and Moss per sample in the AML dataset. 11
S12 Common SNVs called by Strelka2 and Moss per sample in the CRC dataset. 11
S13 Results of Moss with Strelka2 on patient 45 of the colorectal cancer dataset 12

A Additional results

All experiments were run on a machine with two 64 bit x86 Intel Xeon E5-2698 v4 CPUs at 2.20 GHz and 512 GB of memory.

- Precision-recall curves in Figure S1 show that Moss improves the F_1-score on the simulated datasets with 30× and 60× depth when using $m \in \{2, 3, 4\}$ samples with respect to running the single callers Mutect2 [2] and Strelka2 [6] in isolation and the multi-sample callers multisnv [5] and multi-sample Mutect2. When run in conjunction with the single-callers Mutect2 and Strelka2, Moss improves the recall without loss in precision in all cases. Results for multi-sample callers mutisnv and multi-sample Mutect2 are also shown. Moss run in conjunction with Strelka2 achieves the highest F_1 score in all cases.

- Figure S2 shows that Moss incurs a small overhead in running time when compared to the single-sample callers Mutect2 and Strelka2. Running Moss after Mutect2 and Strelka2 increases the run time by 9.57% and 21.2%, respectively. Moreover, running Moss in conjunction with single-sample Mutect2 or Strelka2 is more efficient than multi-sample Mutect2 and multisnv.

- Figure S3a compares the call set of Strelka2 when run in isolation and when run in conjunction with Moss on the HCC dataset [7]. Moss identifies 490 new variants while retaining almost all variants identified by Strelka2. Figure S3b shows the number of supporting samples identified by Moss (x-axis) and the single-sample caller Strelka2 (y-axis) for each variant, showing that Moss increases the number of supporting samples for 23% of variants (1,264 variants). Variants recovered by Moss correspond to entries with y-axis equal to 0. Figure S3c compares exposure to mutational signatures of liver tumor for the different methods. Applying Moss increases the total exposure from 52.3% to 55.9%. Figure S3d is a scatter plot showing the number of samples identified by Moss to contain a variant as a function of the variant's largest frequency across all tumor samples. Most of the variants recovered by Moss have low VAF. In Figure S3e, we show that analyzing multiple-samples simultaneously increases the number of recovered variants significantly.

- In the HCC dataset [7], Moss misses a variant called by Mutect2 at chromosome 12 position 125,397,891 when run in conjunction with Mutect2. The mutated base of that SNV appears in 3 samples: A66, B33, and D54. The distribution of base call qualities in each sample are shown in Figure S4 with blue bars representing the normal allele and orange bars representing the mutated allele. The base qualities of the mutated allele are significantly lower than those of the normal allele, which lead to a somatic SNV probability below the *pass* threshold for Moss.

- In Figure S5, we show that Moss increases the number of variants identified in more than one spatial region in the hepatocellular carcinoma (HCC) dataset [7].

- In Figure S6, we show the number of samples identified by Moss to contain a variant as a function of the variant's largest frequency in the normal sample. It shows that the recovered SNVs by Moss (green dots) have a low VAF in the normal sample (no more than 0.06), which suggests that the recovered SNVs are not present in the germline.

- In Figure S7, we show the result of manual review of SNVs in HCC data [7] called by Moss in $m = 2$ samples and not called by Mutect2 in any sample.

- Figure S8 shows that Moss can still recover variants on low-coverage data even when only few samples are available.

- Figure S9 shows that compared to the single-sample caller, the increase in run time and memory usage of Moss is negligible. Running Moss on top of Mutect2 and Strelka2 increases the run time by 1.8% and 6.4%, respectively, and its memory consumption is less than 400 MB.

- In the AML dataset [3], Moss misses a variant called by Mutect2 that is contained in the gold list. We show in Figure S10 the distribution of base call qualities in each sample for the normal allele (blue bars) and the mutated allele (orange bars). The base qualities of the mutated allele are significantly lower than those of the normal allele, and the SNV is filtered out by Moss.

- In Figure S11, we show that Moss increases the number of variants identified in more than one temporal sample in the acute myeloid leukymia (AML) dataset [3].
- In Figure S12, we show that Moss increases the number of variants identified in more than one sample in the hepatocellular carcinoma (HCC) dataset.
- In Figure S13 we show the results of Moss with Strelka2 on patient 45 of the colorectal cancer (CRC) dataset [4]. Moss recovers 40,234 SNVs, while losing 17 variants called by Strelka2. The number of supporting samples is further increased for 33% of variants (68,661 variants). The composition of colorectal signatures remains the same with the recovered variants, but the total exposure is increased.

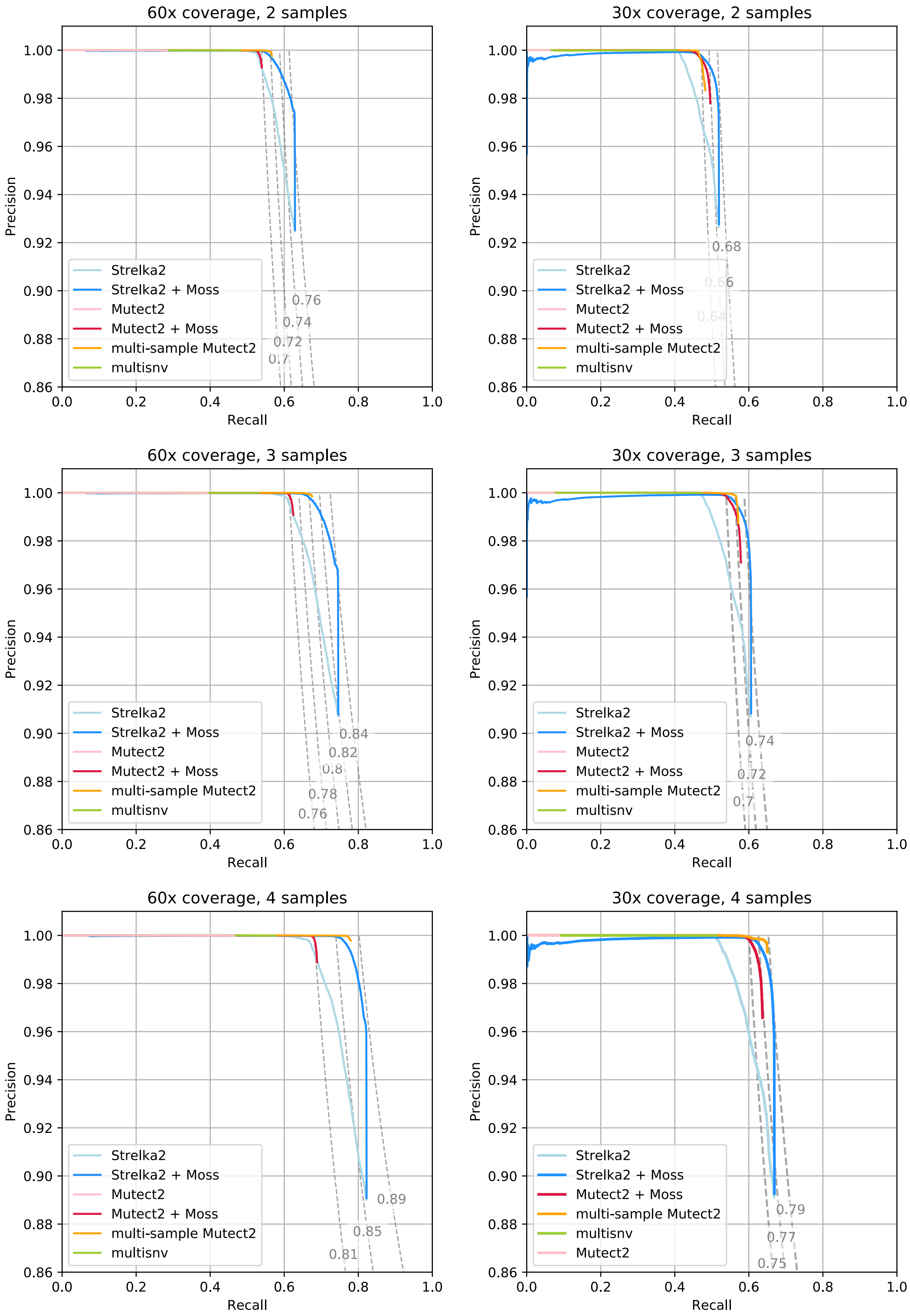

Figure S1: **Moss improves performance on simulated data on 2, 3, and 4 tumor samples.** Precision-recall curves of the union of SNVs identified by Strelka2 (light blue) and Mutect2 (light red) when applied to single samples of a simulated bulk DNA sequencing dataset (with $m \in \{2, 3, 4\}$ samples and 30x and 60x coverage), as well as when Moss is applied in conjunction with these methods (blue and red). Results of two other multi-sample SNV methods, multisnv (light green) and multi-sample Mutect2 (orange), are also listed for comparison. The dashed lines represent F_1 score isolines (i.e., harmonic mean between recall and precision). In all cases, Moss improves the recall without loss in precision. Single-sample Mutect2 is from GATK v4.0.12; multi-sample Mutect2 is from GATK v4.1.7.

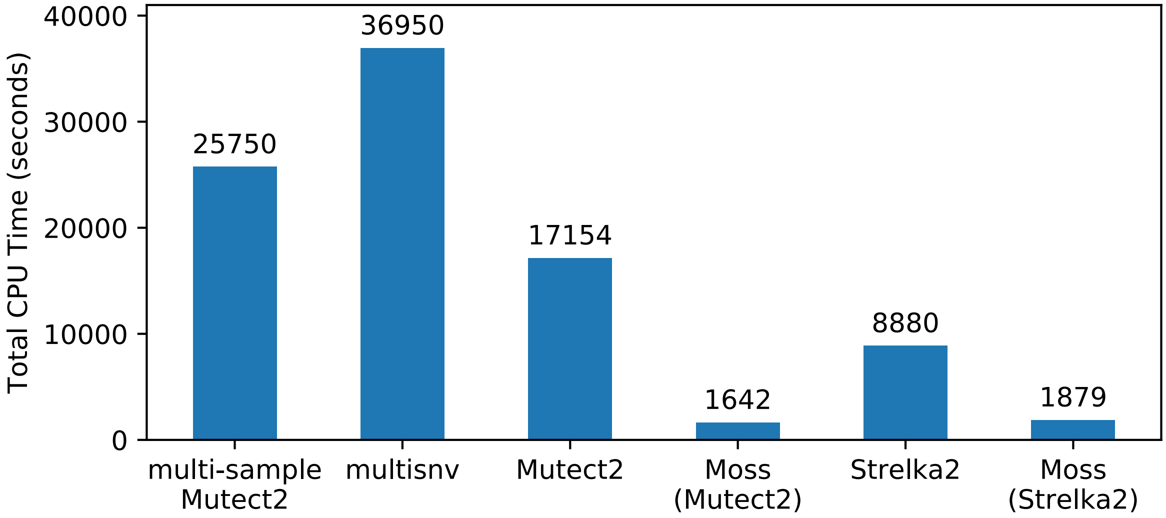

Figure S2: **Moss incurs a small overhead in run time in the simulated dataset. Running multi-sample Mutect2 and multisnv takes more time than running Moss in conjunction with single-sample callers Mutect2 or Strelka2.** Comparison of run time of multisnv, both multi-sample and single-sample Mutect2, Strelka2 and Moss on the simulated dataset with 5 tumor samples and a coverage of $60\times$. Running Moss on top of Mutect2 and Strelka2 increases the run time by 9.57% and 21.2%, respectively. The total CPU time for Moss is measured with the "time" command in Linux. Single-sample Mutect2 is from GATK v4.0.12; multi-sample Mutect2 is from GATK v4.1.7.

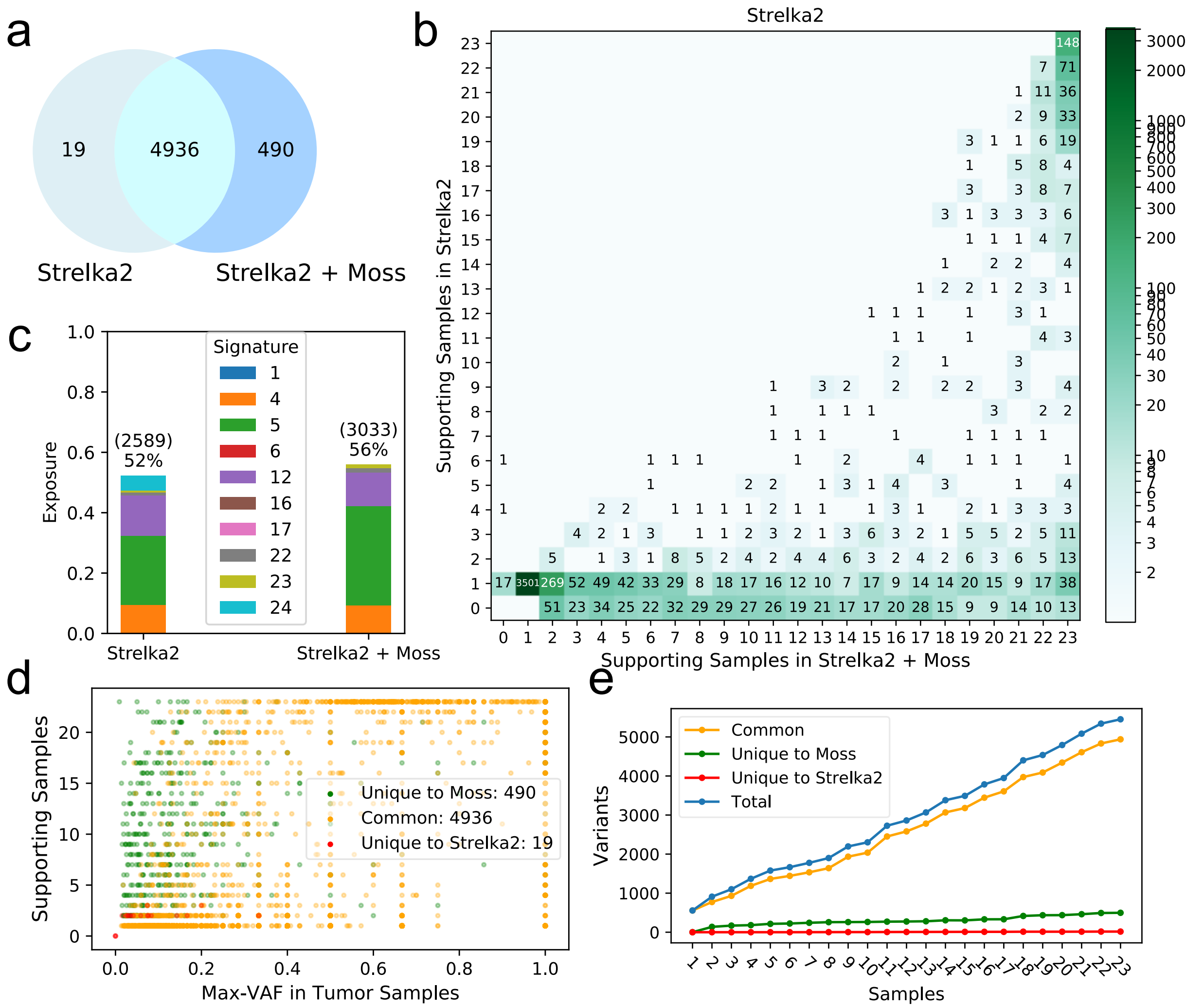

Figure S3: **Moss recovers high quality somatic variants missed by Strelka2 in the hepatocellular carcinoma (HCC) dataset [7].** (a) Venn diagram comparing the call set of Strelka2 when run in isolation and when run in conjunction with Moss. Moss identifies 490 new variants while retaining almost all variants identified by Strelka2. (b) The number of supporting samples identified by Moss (x-axis) and the single-sample caller Strelka2 (y-axis) for each variant, showing that Moss increases the number of supporting samples for 23% of variants (1,264 variants). Variants recovered by Moss correspond to entries with y-axis equal to 0. (c) Exposure to mutational signatures of liver tumor for the different methods. Applying Moss results in a slightly higher total exposure (52.3% vs 55.9%) and a higher number of SNVs explained by related signatures (2,589 to 3,033). (d) The number of samples identified by Moss to contain a variant as a function of the variant's largest frequency across all tumor samples, showing that most of the variants recovered by Moss have low VAF. Color indicates whether the variant is common to Strelka2 and Moss (yellow), or unique to Moss (green) or Strelka2 (red). (e) Analyzing multiple-samples simultaneously increases the number of recovered variants significantly. The randomized order is: C2, B9, B6, B45, B5, A25, A66, C3, A5, D62, B33, C31, A58, D29, C74, D25, A61, Z1, D16, B4, D54, D58, D63. This order was also used for the same test with Mutect2 (main text Fig. 3g) and the low-coverage data (Supplementary Fig. S8).

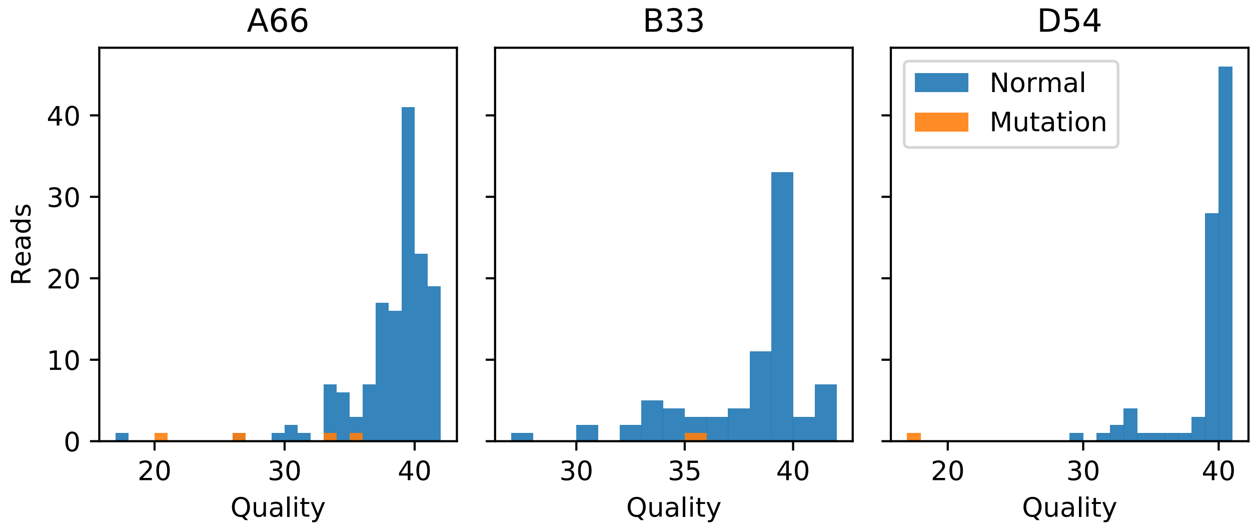

Figure S4: **The reads supporting the one SNV from the HCC data [7] missed by Moss and called by Mutect2 have lower base quality compared to reads supporting the normal allele.** Distribution of base call qualities of the samples supporting the SNV called by Mutect2 and lost when running Moss in the hepatocellular carcinoma (HCC) dataset (main text Fig. 3b). The SNV is located in chromosome 12 at position 125,397,891. The mutated bases (orange) only appear in 3 of the samples (A66, B33 and D54), and they exhibit lower qualities as compared to the non-mutated bases (blue).

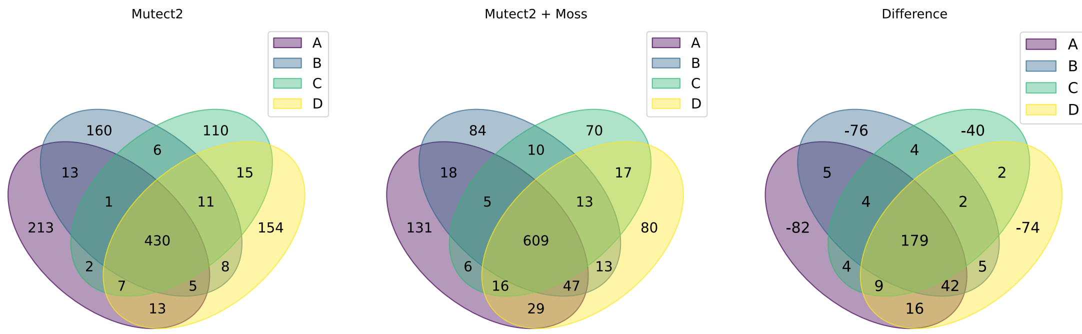

Figure S5: **Moss increases the number of variants identified in more than one spatial region on the hepatocellular carcinoma (HCC) dataset [7].** This analysis is restricted to the 1,148 SNVs detected by both Mutect2 and Moss. The $m = 23$ samples are grouped into four quadrants (A, B, C and D) as defined in [7]. Sample 'Z1' was included in quadrant C. For each common variant, we indicate in which quadrants it was detected by Mutect2 (left) or Mutect2 + Moss (middle). The right Venn diagram shows that Moss increases the number of common identified variants that intersect in at least 2 quadrants.

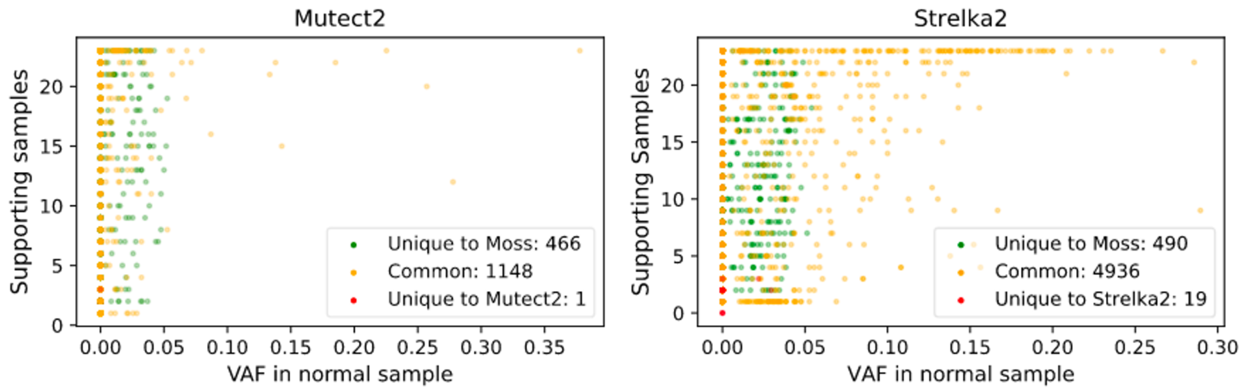

Figure S6: **SNVs recovered by Moss in the HCC data [7] have low VAFs in the matched normal sample.** We show for each called variant the number of supporting samples identified by Moss as a function of the variant's frequency in the normal sample, showing that variants recovered by Moss have low VAF in the normal sample (no more than 0.06).

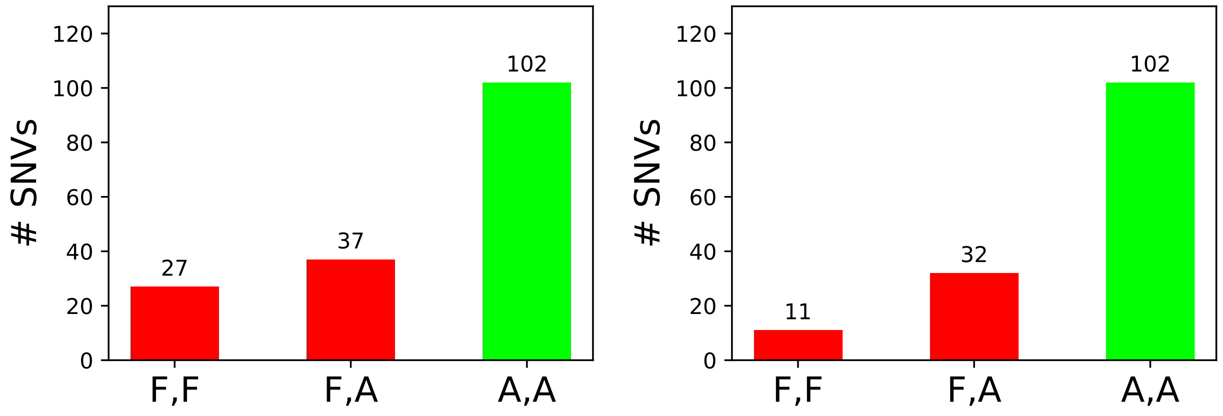

(a) All 166 variants prior to filtering

(b) Variants called by Moss after filtering

Figure S7: **Manual review of SNVs in HCC data [7] called by Moss in $m = 2$ samples and not called by Mutect2 in any sample.** Annotations of (a) all variants called by Moss in 2 samples and not identified by Mutect2 in any sample prior to filtering, and of (b) retained variants after applying Moss' filters. When analyzing a given variant, we look at the specific locus in both samples in isolation, and label them in each sample as Somatic (S), Germline (G), Ambiguous (A) (for variants that look correct) or Failed (F) following the manual review protocol [1]. None of the 166 variants were designated as 'G' or 'S'. While variants indicated as 'F' are sequencing/mapping artifacts, variants indicated as 'A' lack strong evidence to be classified as either a correct variant or an artifact. Variants labeled as 'A' in both samples are likely correct.

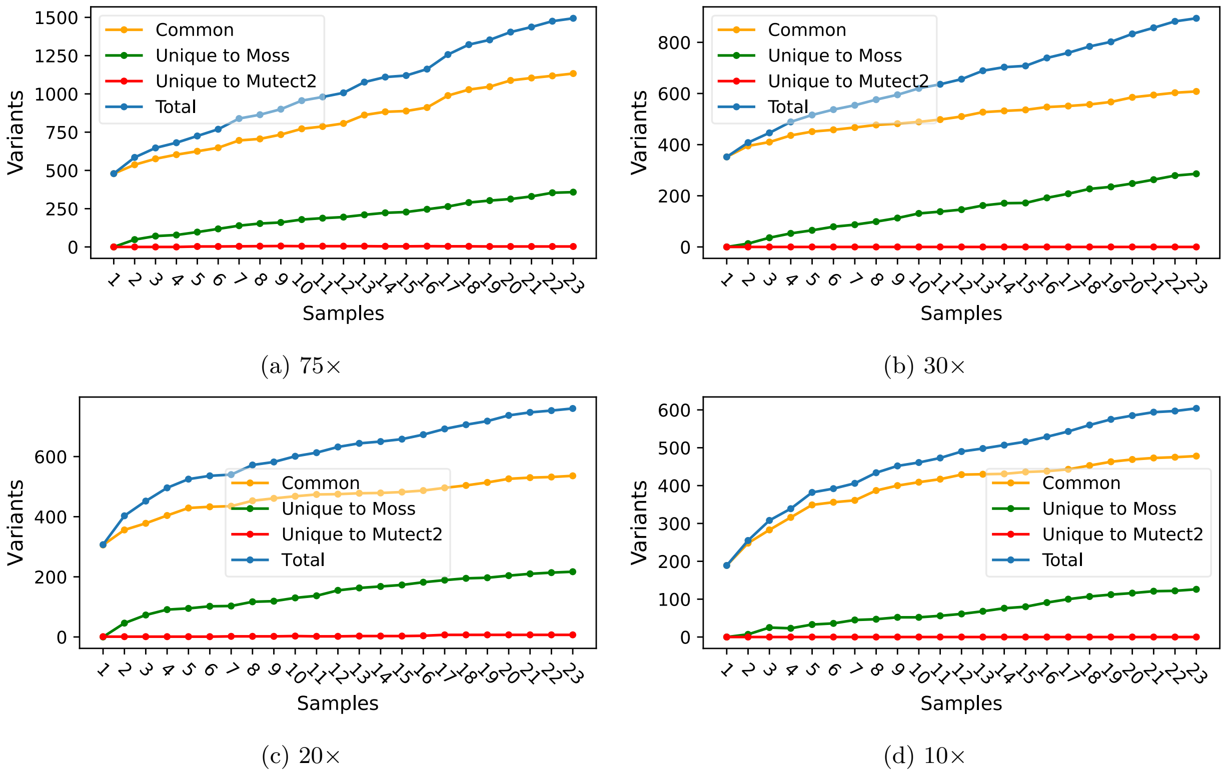

(a) 75×

(b) 30×

(c) 20×

(d) 10×

Figure S8: **Moss is able to recover variants on low-coverage data.** In the HCC dataset [7], analyzing multiple-samples simultaneously increases the number of recovered variants significantly (as compared to running Mutect2 independently in each sample), both in the (a) original data and when downsampled to (b) 30×, (c) 20×, and (d) 10×. The total number of called variants in the downsampled data decreases as compared to the original one, but the trend is the same.

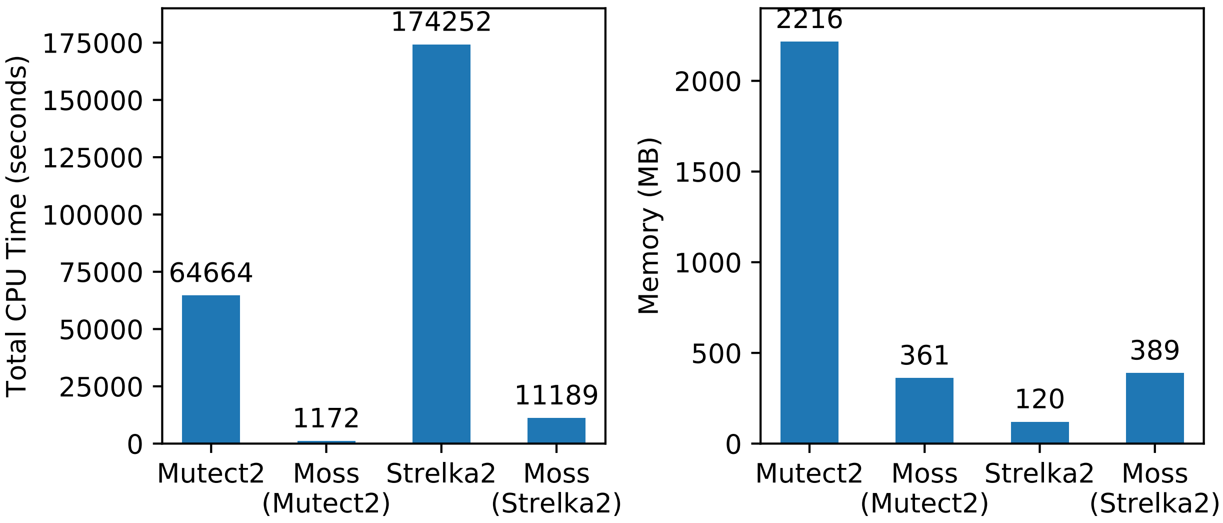

Figure S9: **Moss incurs almost no overhead in run time, and has memory consumption of less than 400MB in the HCC dataset [7].** Comparison of run time and memory usage of Mutect2, Strelka2 and Moss on the hepatocellular carcinoma (HCC) dataset. Running Moss on top of Mutect2 and Strelka2 increases the run time by 1.8% and 6.4%, respectively, and its memory consumption is less than 400 MB. The total CPU time and the memory for Moss is measured with the “time” command in Linux, and we use the maximum memory usage logged by Mutect2. Moss filters are applied on rediscovered variants. MB: megabyte.

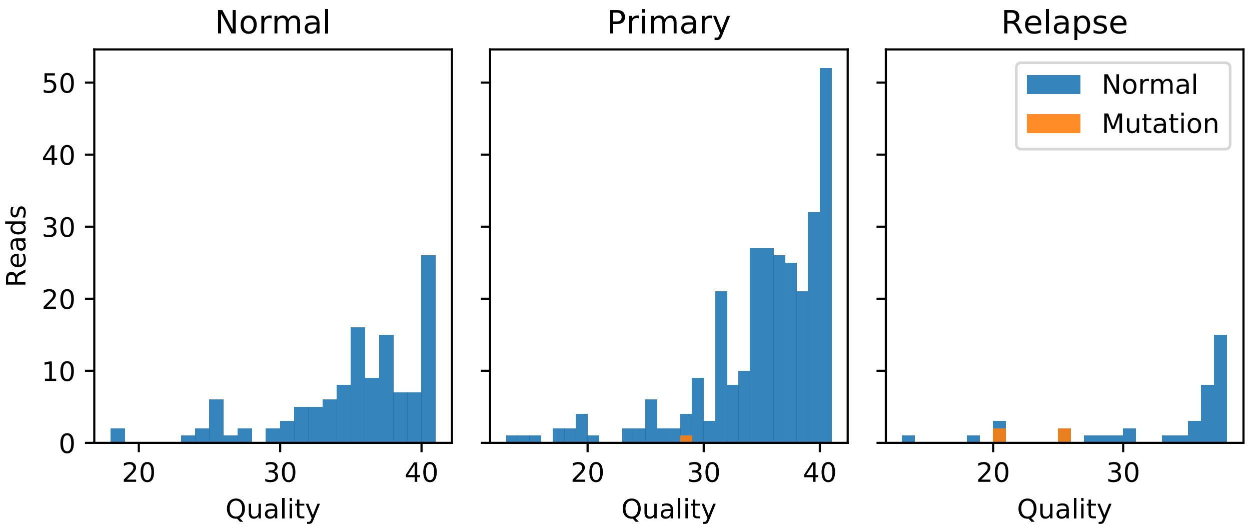

Figure S10: **The reads supporting the one SNV from the AML data [3] missed by Moss and called by Mutect2 have lower base quality compared to reads supporting the normal allele.** Distribution of base call qualities of the samples supporting the SNV called by Mutect2 and lost when running Moss in the acute myeloid leukimia (AML) dataset (main text Fig. 4b). The SNV is located in chromosome 5 at position 38,141,127. The mutated bases (orange) appeared 1 time and 4 times in the primary and relapse sample, respectively, and they exhibit lower qualities as compared to the non-mutated bases (blue).

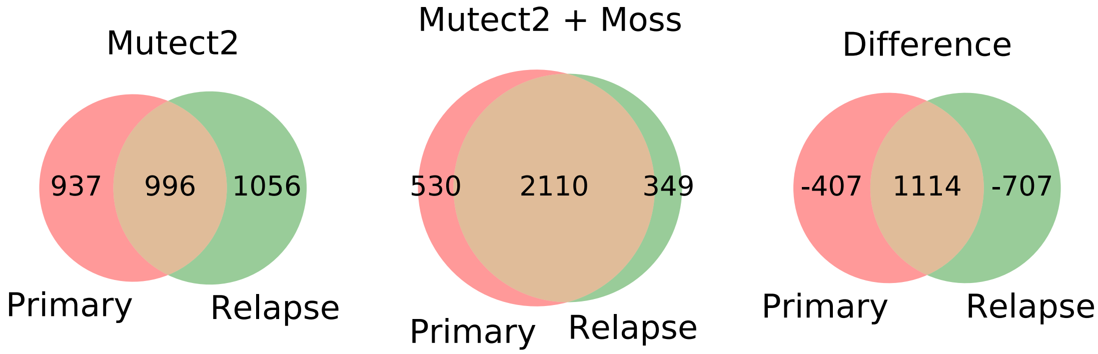

Figure S11: **Moss increases the number of variants identified in both the primary tumor and relapse samples on the acute myeloid leukemia (AML) data [3].** This analysis is restricted to the 2,989 SNVs detected by both Mutect2 and Moss. For each common variant, we indicate in which sample it was detected by Mutect2 (left) or Mutect2 + Moss (middle). The right Venn diagram shows that Moss increases the number of common identified variants that are present in both samples.

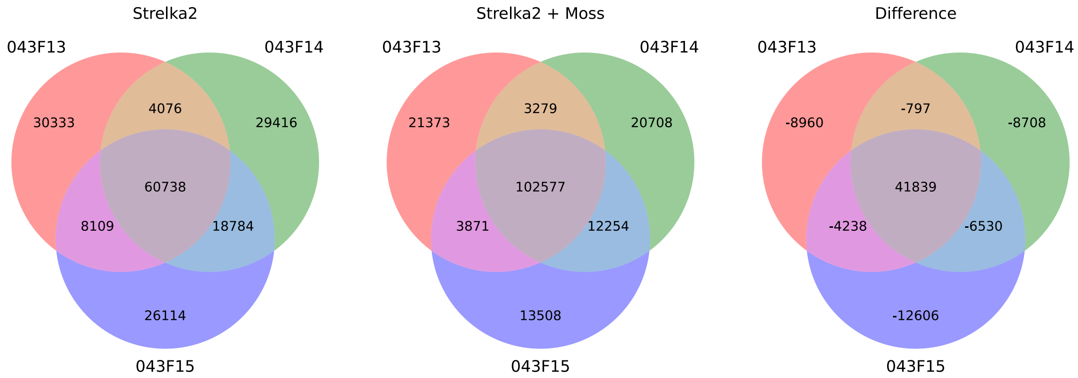

(a) Patient 43

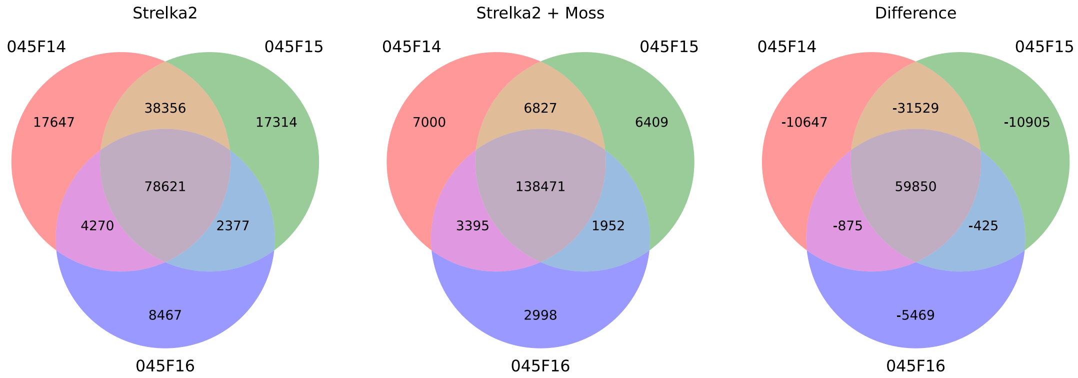

(b) Patient 45

Figure S12: **Moss increases the number of variants identified in more than one sample on the the colorectal cancer (CRC) data [4].** This analysis is restricted to the SNVs detected by both Strelka2 and Moss in *Patient 43* (177,570 SNVs) and *Patient 45* (167,052 SNVs). For each common variant, we indicate in which samples it was detected by Strelka2 (left) or Strelka2 + Moss (middle). The right Venn diagram (for both patients) shows that Moss increases the number of common identified variants that are present in at least two samples.

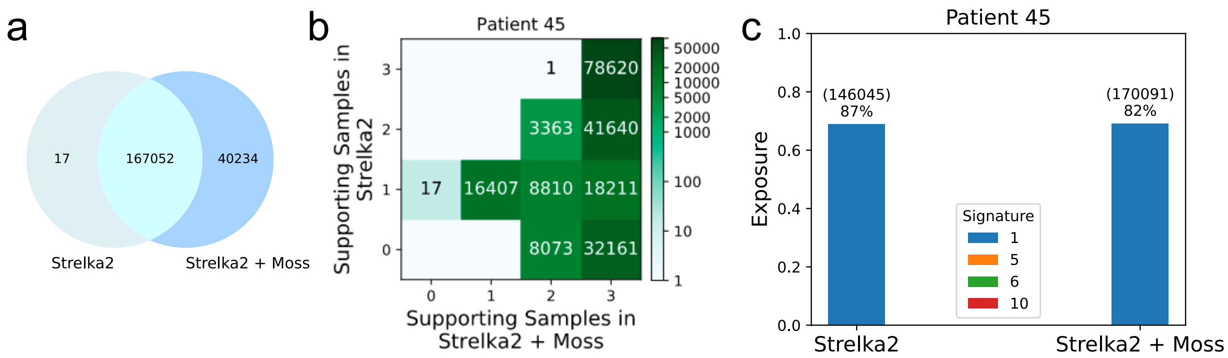

Figure S13: **Moss recovers high quality somatic variants missed by Strelka2 on patient 45 of the colorectal cancer (CRC) dataset [4].** (a) Venn diagram comparing the call set of Strelka2 when run in isolation and when run in conjunction with Moss. Moss identifies 40,234 new variants while retaining almost all variants identified by Strelka2. (b) The number of supporting samples by Moss (x-axis) and the single-sample caller Strelka2 (y-axis) for each variant, showing that Moss increases the number of supporting samples for 33% of variants (68,661 variants). (c) Exposure to mutational signatures of colorectal tumor for the different methods. The identified signatures remain the same, but the exposure is increased with Moss.

B Implementation details

Efficient calculation of the somatic probability. Calculating the complement of the *somatic probability* (Equation (1)) requires summing over all possible 2^m combinations of $\mathbf{z}$, which grows exponentially with the number of samples and hence does not scale.

$$\begin{aligned}\mathbf{P}(\mathbf{Z}=\mathbf{0}\,|\,\mathcal{N},\mathbf{b},\mathbf{q}) &= \frac{\mathbf{P}(\mathbf{b}\,|\,\mathbf{Z}=\mathbf{0},\mathcal{N},\mathbf{q})\mathbf{P}(\mathbf{Z}=\mathbf{0})}{\sum_{\mathcal{T}\neq\mathcal{N}}\sum_{\mathbf{z}\in\{0,1\}^m}\mathbf{P}(\mathbf{b},\mathbf{Z}=\mathbf{z},\mathcal{T}\,|\,\mathcal{N},\mathbf{q})} \\ &= \frac{\sum_{\mathcal{T}\neq\mathcal{N}}\mathbf{P}(\mathbf{b}\,|\,\mathbf{Z}=\mathbf{0},\mathcal{T},\mathcal{N},\mathbf{q})\mathbf{P}(\mathbf{Z}=\mathbf{0})}{\sum_{\mathcal{T}\neq\mathcal{N}}\sum_{\mathbf{z}\in\{0,1\}^m}\mathbf{P}(\mathbf{b}\,|\,\mathbf{Z}=\mathbf{z},\mathcal{T},\mathcal{N},\mathbf{q})\mathbf{P}(\mathbf{Z}=\mathbf{z})}\end{aligned} \tag{1}$$

We have the following prior for $\mathbf{P}(\mathbf{Z}=\mathbf{z})$, which is uniform except for $\mathbf{z}=\mathbf{0}$.

$$\mathbf{P}(\mathbf{Z}=\mathbf{z}) = \begin{cases} 1-\mu, & \text{if } \mathbf{z}=\mathbf{0}, \\ \dfrac{\mu}{2^m-1}, & \text{if } \mathbf{z}\neq\mathbf{0}. \end{cases} \tag{2}$$

Given the prior for $\mathbf{P}(\mathbf{Z}=\mathbf{z})$ (Equation (2)), we can recombine the terms, bringing down the time complexity from $O(2^m)$ to $O(m)$ as follows:

$$\begin{aligned}&\sum_{\mathbf{z}\in\{0,1\}^m}\mathbf{P}(\mathbf{b}\,|\,\mathbf{Z}=\mathbf{z},\mathcal{T},\mathcal{N},\mathbf{q})\mathbf{P}(\mathbf{Z}=\mathbf{z}) \\ =&\sum_{\mathbf{z}\in\{0,1\}^m}\mathbf{P}(\mathbf{Z}=\mathbf{z})\prod_{i=1}^m\mathbf{P}(\mathbf{b}_i\,|\,Z_i=z_i,\mathcal{T},\mathcal{N},\mathbf{q}_i) \\ =&(1-\mu)\prod_{i=1}^m\mathbf{P}(\mathbf{b}_i\,|\,Z_i=0,\mathcal{T},\mathcal{N},\mathbf{q}_i)+\sum_{\mathbf{z}\in\{0,1\}^m\setminus\{\mathbf{0}\}}\frac{\mu}{2^m-1}\prod_{i=1}^m\mathbf{P}(\mathbf{b}_i\,|\,Z_i=z_i,\mathcal{T},\mathcal{N},\mathbf{q}_i) \\ =&\left(1-\frac{2^m}{2^m-1}\mu\right)\prod_{i=1}^m\mathbf{P}(\mathbf{b}_i\,|\,Z_i=0,\mathcal{T},\mathcal{N},\mathbf{q}_i)+\frac{\mu}{2^m-1}\sum_{\mathbf{z}\in\{0,1\}^m}\prod_{i=1}^m\mathbf{P}(\mathbf{b}_i\,|\,Z_i=z_i,\mathcal{T},\mathcal{N},\mathbf{q}_i)\end{aligned} \tag{3}$$

Let $p_i^0=\mathbf{P}(\mathbf{b}_i\,|\,Z_i=0,\mathcal{T},\mathcal{N},\mathbf{q}_i)$ and $p_i^1=\mathbf{P}(\mathbf{b}_i\,|\,Z_i=1,\mathcal{T},\mathcal{N},\mathbf{q}_i)$. Then,

$$\sum_{\mathbf{z}\in\{0,1\}^m}\prod_{i=1}^m\mathbf{P}(\mathbf{b}_i\,|\,Z_i=z_i,\mathcal{T},\mathcal{N},\mathbf{q}_i)=\sum_{\mathbf{z}\in\{0,1\}^m}\prod_{i=1}^m p_i^{z_i}=\prod_{i=1}^m\left(p_i^0+p_i^1\right), \tag{4}$$

and Equation (3) becomes

$$\left(1-\frac{2^m}{2^m-1}\mu\right)\prod_{i=1}^m p_i^0+\frac{\mu}{2^m-1}\prod_{i=1}^m(p_i^0+p_i^1), \tag{5}$$

with complexity $O(m)$.

Omission of samples. Fixing the number of samples containing bases different from the normal allele, the somatic probability (Equation (1)) converges to 0 with an increasing number of all-normal samples (i.e., samples for which all bases are equal to the normal allele $\mathcal{N}$). Here, we show that by omitting these all-normal samples from the computation, the somatic probability does not converge to either zero or one. Hence by default Moss excludes the all-normal samples in the computation of the somatic probability.

To understand why this adjustment is required, consider the case where there are m tumor samples. A subset of k samples contain bases different from the normal allele $\mathcal{N}$, and the remaining $m-k$ samples contain only bases equal to $\mathcal{N}$ (i.e., all-normal samples). For simplicity, assume that all bases (for all samples) have the same error probability q associated to them, and that all samples have the same depth. Further assume that the non-normal bases are identical. Also, assume that the number of non-normal bases is the same across all samples which contain them. We denote by $\mathbf{b}_\mathcal{T}$ the bases of a tumor sample containing at least

one base different from $\mathcal{N}$, and by $\mathbf{b}_\mathcal{N}$ the bases of a tumor sample containing only bases equal to $\mathcal{N}$. We define the following four probabilities.

$$\alpha = \sum_{\mathcal{T}\neq\mathcal{N}} \mathbf{P}(\mathbf{b}_\mathcal{T} \,|\, z_i = 0, \mathcal{T}, \mathcal{N}, q),$$
$$\beta = \sum_{\mathcal{T}\neq\mathcal{N}} \mathbf{P}(\mathbf{b}_\mathcal{T} \,|\, z_i = 1, \mathcal{T}, \mathcal{N}, q),$$
$$\gamma = \mathbf{P}(\mathbf{b}_\mathcal{N} \,|\, z_i = 0, \mathcal{T}, \mathcal{N}, q) = (1-q)^{|\mathbf{b}_\mathcal{N}|},$$
$$\delta = \mathbf{P}(\mathbf{b}_\mathcal{N} \,|\, z_i = 1, \mathcal{T}, \mathcal{N}, q) = \int_0^1 \Big(f_i \frac{q}{3} + (1-f_i)(1-q)\Big)^{|\mathbf{b}_\mathcal{N}|} \mathbf{P}(f_i \,|\, z_i)\,\mathrm{d}f_i,$$

where α and β indicate the likelihood of $\mathbf{b}_\mathcal{T}$ given $z_i = 0$ and $z_i = 1$, respectively, as well as the normal and the quality scores marginalized over all possible tumor alleles. Similarly, γ and δ indicate the likelihood of $\mathbf{b}_\mathcal{N}$ given $z_i = 0$ and $z_i = 1$, respectively, as well as the normal and the quality scores. Note that γ and δ are actually independent of the tumor allele $\mathcal{T}$. Therefore, α and β can be directly multiplied with γ or δ. Note also that we have replaced $\mathbf{q}$ with q due to the assumption that all quality scores equal q for all samples and all bases.

Consider the prior for $\mathbf{Z}$ specified in Equation (2), and let the number of samples $m \to \infty$ (for a fixed constant k). We have

$$\begin{aligned}
\lim_{m\to\infty} \mathbf{P}_m &= \lim_{m\to\infty} \mathbf{P}(\mathbf{Z} = \mathbf{0} \,|\, \mathcal{N}, \mathbf{b}, \mathbf{q}) \\
&= \lim_{m\to\infty} \frac{\sum_{\mathcal{T}\neq\mathcal{N}} \prod_{i=1}^m \mathbf{P}(\mathbf{b}_i \,|\, z_i = 0, \mathcal{T}, \mathcal{N}, q)(1-\mu)}{\sum_{\mathbf{z}\in\{0,1\}^m} \mathbf{P}(\mathbf{z} \,|\, \mathcal{N}) \sum_{\mathcal{T}\neq\mathcal{N}} \prod_{i=1}^m \mathbf{P}(\mathbf{b}_i \,|\, z_i, \mathcal{T}, \mathcal{N}, q)} \\
&= \lim_{m\to\infty} \frac{\alpha^k \gamma^{m-k}(1-\mu)}{\alpha^k\gamma^{m-k}(1-\mu) + \Big((\alpha+\beta)^k(\delta+\gamma)^{m-k} - (\alpha+\beta)^k\gamma^{m-k}\Big)\frac{\mu}{2^m-1} + \Big((\alpha+\beta)^k - \alpha^k\Big)\gamma^{m-k}\frac{\mu}{2^m-1}} \\
&= \lim_{m\to\infty} \frac{\alpha^k(1-\mu)}{\alpha^k(1-\mu) + (\alpha+\beta)^k\frac{(\delta+\gamma)^{m-k}-\gamma^{m-k}}{\gamma^{m-k}}\frac{\mu}{2^m-1} + \Big((\alpha+\beta)^k - \alpha^k\Big)\frac{\mu}{2^m-1}} = 1
\end{aligned}$$

Hence, for a constant number k of samples that contain bases different from $\mathcal{N}$, the somatic probability $1 - \mathbf{P}_m$ converges to zero as the number of all-normal samples increases. Note that the term $(\alpha+\beta)^k\frac{(\delta+\gamma)^{m-k}-\gamma^{m-k}}{\gamma^{m-1}}\frac{\mu}{2^m-1}$ converges to zero as expected, since it corresponds to having a non-zero z in at least one all-normal sample. However, the term $\Big((\alpha+\beta)^k - \alpha^k\Big)\frac{\mu}{2^m-1}$, which corresponds to having an SNV in at least one sample among the k tumor samples that are not all-normal, also decreases to 0 exponentially. Hence the considered prior for $\mathbf{Z}$ causes the somatic probability to converge to zero, regardless of the value of k and the observed variant allele frequency (VAF).

Modifying the prior to be independent for each sample as $\mathbf{P}(\mathbf{Z} = \mathbf{z}) = \prod_{i=1}^m (1-\mu)^{\mathbb{1}\{z_i=0\}}\mu^{\mathbb{1}\{z_i=1\}}$, in addition to being unrealistic, does not solve the problem. In particular, $\mathbf{P}_m$ converges to 0 in this case and the somatic probability goes to 1, independently of k.

We now consider our proposed solution. That is, we consider the case in which the all-normal samples are ignored for the computation of the somatic probability, using the joint prior for $\mathbf{Z}$ given in Equation (2). In this case we consider $\mathbf{Z} \in \{0,1\}^k$, obtaining

$$\begin{aligned}
\mathbf{P}(\mathbf{Z} = \mathbf{0} \,|\, \mathcal{N}, \mathbf{b}, \mathbf{q}) &= \frac{\sum_{\mathcal{T}\neq\mathcal{N}} \prod_{i=1}^k \mathbf{P}(\mathbf{b}_i \,|\, z_i = 0, \mathcal{T}, \mathcal{N}, q)(1-\mu)}{\sum_{\mathbf{z}\in\{0,1\}^k} \mathbf{P}(\mathbf{z} \,|\, \mathcal{N}) \sum_{\mathcal{T}\neq\mathcal{N}} \prod_{i=1}^k \mathbf{P}(\mathbf{b}_i \,|\, z_i, \mathcal{T}, \mathcal{N}, q)} \\
&= \frac{\alpha^k(1-\mu)}{\alpha^k(1-\mu) + \Big((\alpha+\beta)^k - \alpha^k\Big)\frac{\mu}{2^k-1}},
\end{aligned}$$

which does not depend on m. Hence Moss by default ignores the samples that contain all bases equal to the $\mathcal{N}$ allele for the computation.

C Commands

C.1 Strelka2

1. Generate Python script using the given configuring script with the default settings.

```
python2 bin/configureStrelkaSomaticWorkflow.py \
    --tumorBam ${tumor} \
    --normalBam ${normal} \
    --referenceFasta ${ref} \
    --runDir ${dir}
```

2. Run the generated Python script.

```
python2 ${dir}/runWorkflow.py -m local -j 10
```

C.2 Mutect2

```
${gatk} Mutect2 \
    -R ${ref} \
    -I ${input} \
    -I ${normal} \
    -tumor ${sample_name} \
    -normal normal \
    --germline-resource ${gnomad} \
    --af-of-alleles-not-in-resource 0.0000025 \
    --disable-read-filter MateOnSameContigOrNoMappedMateReadFilter \
    -L ${bed} \
    -O ${output} \
    -bamout ${input}.mutect2.bam

${gatk} GetPileupSummaries \
    -I ${input} \
    -V small_exac_common_3.hg38.vcf.gz \
    -L small_exac_common_3.hg38.vcf.gz \
    -O ${input}.pileup.table

${gatk} CalculateContamination \
    -I ${input}.pileup.table \
    -matched ${normal}.pileup.table \
    -O ${input}.contamination.table

${gatk} FilterMutectCalls \
    -V ${output} \
    --contamination-table ${input}.contamination.table \
    -O ${output}.filtered.vcf.gz
```

C.3 Moss

C.3.1 Installation

Moss can be installed either via Conda or built directly from source.

C.3.2 Install Moss via Conda

1. Create a new conda environment named "moss" and install dependencies:

   ```
   conda create -n moss
   ```

2. Then activate the created environment:

   ```
   conda activate moss
   ```

3. Install the package into the current environment "moss":

   ```
   conda install -c bioconda moss
   ```

C.3.3 Build Moss from source

Dependencies. Moss is written in C++11 and thus requires a modern C++ compiler (GCC $\geq$ 4.8.1, or Clang). In addition, Moss has the following dependencies:

- HTSlib ($\geq$ 1.7)
- CMake ($\geq$ 3.9)
- Python ($\geq$ 3.6)
- Scikit-allel

Compilation. To compile Moss, use the following commands.

```
mkdir build
cd build
cmake -DCMAKE_BUILD_TYPE=Release ..

# If HTSlib is not in the system path, CMake may not be able to find it.
# Users then need to manually set the path for htslib using ccmake:
ccmake ..

# Then finally make Moss.
make
```

The compilation results in the executable `moss`.

C.4 Usage

1. Generating the list of candidates:

   ```
   python scripts/union_candidates.py -f list_of_VCF.list --normal-name NORMAL \
       -t Mutect -o candidates.chrdemo20.vcf
   ```

   Note that we remove variants flagged as "*artifact in normal*" by Mutect2.

2. Run Moss:

```
./moss -r ../data/demo20.fa -b ../data/normal.sort.bam \
    -b ../data/clone0.spike.sort.bam \
    -b ../data/clone1.spike.sort.bam \
    -b ../data/clone2.spike.sort.bam \
    -b ../data/clone3.spike.sort.bam \
    -l ../data/candidates.chrdemo20.vcf \
    -m 4 -t -0.693 --ignore0 --grid-size 200 \
    -o example.vcf
```

3. Post-filtering:

```
tabix
python scripts/post_filter.py -i candidates.chrdemo20.vcf.gz \
    -o candidates.chrdemo20.post_filter.vcf
```

C.4.1 Docker usage

Alternatively, Moss can be run as a docker container. The docker image is available on Docker Hub, which can be pulled from Docker Hub by the following command.

```
docker pull chuanyiz/moss
```

Organize your data in the same structure as the `data/` folder. Modify the `config.yaml` following the instructions within the file, and it will be used to generate the commands for Moss within the container. When starting the docker container, you need to bind-mount the data folder by argument `-v path:/moss_data:`, where path represents the **absolute** path to your data folder in the host, and `/moss_data` is the mounted location in the container.

```
docker run -it -v path:/moss_data: chuanyiz/moss /bin/bash \
    -c "python /moss_scripts/run_moss.py  -c /moss_data/config.yaml \
        -o /moss_data/run_moss.sh && bash /moss_data/run_moss.sh"
```

References

[1] Erica K Barnell, Peter Ronning, Katie M Campbell, Kilannin Krysiak, Benjamin J Ainscough, Lana M Sheta, Shahil P Pema, Alina D Schmidt, Megan Richters, Kelsy C Cotto, et al. Standard operating procedure for somatic variant refinement of sequencing data with paired tumor and normal samples. *Genetics in Medicine*, 21(4):972–981, 2019.

[2] David Benjamin, Takuto Sato, Kristian Cibulskis, Gad Getz, Chip Stewart, and Lee Lichtenstein. Calling Somatic SNVs and Indels with Mutect2. *BioRxiv*, page 861054, 2019.

[3] Malachi Griffith, Christopher A Miller, Obi L Griffith, Kilannin Krysiak, Zachary L Skidmore, Avinash Ramu, Jason R Walker, Ha X Dang, Lee Trani, David E Larson, et al. Optimizing cancer genome sequencing and analysis. *Cell systems*, 1(3):210–223, 2015.

[4] Vanessa L Hale, Patricio Jeraldo, Jun Chen, Michael Mundy, Janet Yao, Sambhawa Priya, Gary Keeney, Kelly Lyke, Jason Ridlon, Bryan A White, et al. Distinct microbes, metabolites, and ecologies define the microbiome in deficient and proficient mismatch repair colorectal cancers. *Genome Medicine*, 10(1):78, 2018.

[5] Malvina Josephidou, Andy G Lynch, and Simon Tavaré. multiSNV: a probabilistic approach for improving detection of somatic point mutations from multiple related tumour samples. *Nucleic Acids Research*, 43(9):e61–e61, 2015.

[6] Sangtae Kim, Konrad Scheffler, Aaron L. Halpern, Mitchell A. Bekritsky, Eunho Noh, Morten Källberg, Xiaoyu Chen, Yeonbin Kim, Doruk Beyter, Peter Krusche, and Christopher T. Saunders. Strelka2: fast and accurate calling of germline and somatic variants. *Nature Methods*, 15(8):591–594, 2018.

[7] Shaoping Ling, Zheng Hu, Zuyu Yang, Fang Yang, Yawei Li, Pei Lin, Ke Chen, Lili Dong, Lihua Cao, Yong Tao, et al. Extremely high genetic diversity in a single tumor points to prevalence of non-darwinian cell evolution. *Proceedings of the National Academy of Sciences*, 112(47):E6496–E6505, 2015.
