# Supplementary material for: Moss enables high sensitivity single-nucleotide variant calling from multiple bulk DNA tumor samples
Source: Nat Commun. 2021 Apr 13;12:2204. doi: 10.1038/s41467-021-22466-9 (PMC8044184; doi:10.1038/s41467-021-22466-9)
Supplement: Supplementary file 3 — Description of Additional Supplementary Files [file 41467_2021_22466_MOESM3_ESM.pdf]

Description of Additional Supplementary Files

File Name: Supplementary Data 1
Description: Supplementary Excel file containing the results of the manual review.
