# Supplementary material for: Moss enables high sensitivity single-nucleotide variant calling from multiple bulk DNA tumor samples
Source: Nat Commun. 2021 Apr 13;12:2204. doi: 10.1038/s41467-021-22466-9 (PMC8044184; doi:10.1038/s41467-021-22466-9)
Supplement: Supplementary file 5 — Reporting Summary [file 41467_2021_22466_MOESM5_ESM.pdf]

nature research

Corresponding author(s): Mohammed El-Kebir, Idoia Ochoa

Last updated by author(s): Jan 27, 2021

Reporting Summary

Nature Research wishes to improve the reproducibility of the work that we publish. This form provides structure for consistency and transparency in reporting. For further information on Nature Research policies, see our Editorial Policies and the Editorial Policy Checklist.

Statistics

For all statistical analyses, confirm that the following items are present in the figure legend, table legend, main text, or Methods section.

n/a	Confirmed	
☐	☒	The exact sample size (*n*) for each experimental group/condition, given as a discrete number and unit of measurement
☐	☒	A statement on whether measurements were taken from distinct samples or whether the same sample was measured repeatedly
☒	☐	The statistical test(s) used AND whether they are one- or two-sided<br>*Only common tests should be described solely by name; describe more complex techniques in the Methods section.*
☒	☐	A description of all covariates tested
☐	☒	A description of any assumptions or corrections, such as tests of normality and adjustment for multiple comparisons
☒	☐	A full description of the statistical parameters including central tendency (e.g. means) or other basic estimates (e.g. regression coefficient) AND variation (e.g. standard deviation) or associated estimates of uncertainty (e.g. confidence intervals)
☒	☐	For null hypothesis testing, the test statistic (e.g. *F*, *t*, *r*) with confidence intervals, effect sizes, degrees of freedom and *P* value noted<br>*Give P values as exact values whenever suitable.*
☐	☒	For Bayesian analysis, information on the choice of priors and Markov chain Monte Carlo settings
☒	☐	For hierarchical and complex designs, identification of the appropriate level for tests and full reporting of outcomes
☒	☐	Estimates of effect sizes (e.g. Cohen's *d*, Pearson's *r*), indicating how they were calculated

Our web collection on statistics for biologists contains articles on many of the points above.

Software and code

Policy information about availability of computer code

Data collection	No software was used for data collection.
Data analysis	We developed Moss for identifying low-frequency SNVs that recur in multiple sequencing samples. Moss is open source (MIT license) and publicly available at https://github.com/elkebir-group/Moss. Single sample callers Mutect2 from GATK (v4.0.12) and Strelka2 (v2.9.9) were used to identify SNVs, as well as multi-sample callers Mutect2 from GATK (v4.1.7.) and multisnv (v2.3-14-gb86d4dc). deconstructSig (v1.8.0) was used to calculate the exposure to COSMIC v2 mutational signatures. MASCoTE (https://github.com/raphael-group/mascote, commit: a2dec4f) was used to generate simulated data, which internally uses ART (v2.5.8) for simulating sequencing reads and BWA (v0.7.17) for alignment. BWA-MEM (v0.7.17) was used for alignment, followed by GATK (v4.0.12) for the processing of BAM files of HCC and CRC samples. IGV (v2.4.16) and the add-on IGVNav (commit 8df35a6) were used to perform the manual review.

For manuscripts utilizing custom algorithms or software that are central to the research but not yet described in published literature, software must be made available to editors and reviewers. We strongly encourage code deposition in a community repository (e.g. GitHub). See the Nature Research guidelines for submitting code & software for further information.

Data

Policy information about availability of data

All manuscripts must include a data availability statement. This statement should provide the following information, where applicable:

- Accession codes, unique identifiers, or web links for publicly available datasets
- A list of figures that have associated raw data
- A description of any restrictions on data availability

The analyzed HCC data is available at genome sequence archive of Beijing Institute of Genomics https://bigd.big.ac.cn/gsa/ (accession id PRJCA000091), the AML data is available under accession id dbGaP:phs000159 and the CRC data is available under restricted access, access can be obtained by contacting Nicholas Chia

(Mayo Clinic, Rochester, MN, USA). The generated simulated data is available at https://doi.org/10.13012/B2IDB-9059263_V1.

Field-specific reporting

Please select the one below that is the best fit for your research. If you are not sure, read the appropriate sections before making your selection.

- [x] Life sciences
- [ ] Behavioural & social sciences
- [ ] Ecological, evolutionary & environmental sciences

For a reference copy of the document with all sections, see nature.com/documents/nr-reporting-summary-flat.pdf

Life sciences study design

All studies must disclose on these points even when the disclosure is negative.

Sample size	No experimental data was generated for this work. We analyzed previous published data, which includes HCC data available at genome sequence archive of Beijing Institute of Genomics (https://bigd.big.ac.cn/gsa/, accession id PRJCA000091), the AML data available under accession id dbGaP:phs000159, and the CRC data is not publicly available due to third-party restrictions. The generated simulated data is available at https://doi.org/10.13012/B2IDB-9059263_V1.
Data exclusions	No data were excluded in this study.
Replication	No biological experiments were conducted. The used software is deterministic in nature. As such, all findings in this paper are reproducible with the provided information and input data. For reproducibility, our simulated data are available on Illinois DataBank (https://doi.org/10.13012/B2IDB-9059263_V1) and the used version of Moss to generate the results of this paper is available on Zenodo at https://doi.org/10.5281/zenodo.4487204.
Randomization	Randomization is not relevant to our study, as no experimental data were generated.
Blinding	Blinding is not relevant to our study, as no experimental data were generated.

Reporting for specific materials, systems and methods

We require information from authors about some types of materials, experimental systems and methods used in many studies. Here, indicate whether each material, system or method listed is relevant to your study. If you are not sure if a list item applies to your research, read the appropriate section before selecting a response.

Materials & experimental systems

n/a	Involved in the study	
[x]	[ ]	Antibodies
[x]	[ ]	Eukaryotic cell lines
[x]	[ ]	Palaeontology and archaeology
[x]	[ ]	Animals and other organisms
[x]	[ ]	Human research participants
[x]	[ ]	Clinical data
[x]	[ ]	Dual use research of concern

Methods

n/a	Involved in the study	
[x]	[ ]	ChIP-seq
[x]	[ ]	Flow cytometry
[x]	[ ]	MRI-based neuroimaging
